# Exploring the role of tocotrienol-rich fraction (TRF) in ameliorating neuroinflammation

**DOI:** 10.1007/s10787-026-02249-8

**Published:** 2026-04-20

**Authors:** Jing Yi Tan, Thaarvena Retinasamy, Vanessa Lin Lin Lee, Ammu Kutty Radhakrishnan, Keng Yoon Yeong

**Affiliations:** 1 School of Science, Monash University Malaysia Campus, Jalan Lagoon Selatan, 47500 Bandar Sunway, Selangor Malaysia; 2 Neuropharmacology Research Laboratory, Jeffrey Cheah School of Medicine and Health Sciences, Monash University Malaysia, 47500 Bandar Sunway, Selangor Malaysia; 3 Food As Medicine Research Strength, Jeffrey Cheah School of Medicine and Health Sciences, Monash University Malaysia, 47500 Bandar Sunway, Petaling Jaya, Selangor Malaysia

**Keywords:** Neuroinflammation, Tocotrienol-rich fraction (TRF), Vitamin E, Anti-inflammatory, Antioxidant, Neuroprotection

## Abstract

Neuroinflammation is a chronic inflammatory response that contributes to synaptic dysfunction and neuronal damage, it is a common feature among various neurodegenerative diseases such as Alzheimer’s Disease (AD), Parkinson’s Disease (PD) and Huntington’s Disease (HD). Tocotrienol-rich fraction (TRF) is a form of vitamin E that is known for its anti-inflammatory, antioxidant and neuroprotective properties. Yet, it has not been adequately investigated in both cellular and animal neuroinflammation models. In this study, the potential therapeutic effects of TRF were investigated *in-vitro* using BV2 microglial cells and also *in-vivo* in a pilot study using Sprague Dawley rats. TRF at 5 and 10 µg/mL were found to reduce nitric oxide (NO) and reactive oxygen species (ROS) levels. Furthermore, *in-vivo* treatment with TRF significantly increases the recognition index implying improvement in cognition ability. Gene expression analysis showed downregulation of RelA, TNF-α and IL-6 while NFE2L2 and BDNF were upregulated. These findings suggests that TRF may help mitigates neuroinflammation and oxidative stress, indicating its potential as a candidature for further investigation in neurodegenerative diseases associated with chronic neuroinflammation.

**Supplementary Information:**

The online version contains supplementary material available at 10.1007/s10787-026-02249-8.

## Introduction

In a healthy human, neuroinflammation serves as a crucial defence mechanism for pathogen removal, tissue repair and cellular debris clearance (Kwon and Koh 2020). Once the threat has been successfully eliminated, the immune system returns to its resting state (Zhang et al. 2023). However, persistent inflammatory stimuli due to miscommunication between brain cells will result in chronic neuroinflammation (Adamu et al. 2024; Khoury et al. 2024; Kwon and Koh 2020).

Chronic neuroinflammation promotes a vicious cycle of neuronal damage and degeneration, where an alteration in blood–brain barrier (BBB) permeability allowed immune cells to infiltrate the brain, activating glial cells (microglial and astrocytes) (Adamu et al. 2024; Khoury et al. 2024; Kim and Lee 2024). The activated glial cells then release elevated levels of pro-inflammatory cytokines including interleukin-6 (IL-6) and tumor necrosis factor alpha (TNF-α), along with reactive oxygen species (ROS) and nitric oxide (NO) which in turn trigger multiple molecular signalling pathways. One such pathway is the Nuclear-Factor Kappa B (NF-κB) signalling cascade. Activation of the NF-κB signalling pathway establishes a positive feedback loop that perpetuates pro-inflammatory cytokine, NO and ROS. This process further recruits immune cells that would reactivate the NF-κB signalling pathway, thereby amplifying the inflammatory response and maintaining chronic neuroinflammation (Kim and Lee 2024; Ding et al. 2023; Khoury et al. 2024). This positive feedback loop would cause nitrosative and oxidative stress impeding neuronal regeneration, which leads to synaptic dysfunction and overall neuronal damage (Kwon and Koh 2020; Adamu et al. 2024).

Tocotrienol-Rich Fraction (TRF) is a form of vitamin E that consists of 25% α-tocopherol (α-TCP) and 75% of tocotrienols (Selvaraju et al. 2014a). It can easily pass through the BBB, a criterion which is important for neurotherapies (Fu et al. 2014; Selvaraju et al. 2014b). Moreover, TRF has been shown to activate the brain-derived neurotrophic factor/ tropomyosin receptor kinase B (BDNF/TrKB) pathway and enhancing neuroplasticity (Rui et al. 2024). It also suppresses the activation of the NF-κB pathway, thereby modulating the expression of pro-inflammatory cytokines such as interleukin-1β (IL-1β), IL-6 and TNF-α (Sadikan et al. 2023; Wu et al. 2008). Furthermore, it exhibits antioxidative properties as it upregulates key antioxidant markers including reduced glutathione (GSH) and glutathione peroxidase (GPx), while also functioning as a free radical scavenger (Jayusman et al. 2017; Nor Azman et al. 2018).

Given its critical role in the progression of neurodegenerative diseases, there has been growing scientific interest in developing therapeutic strategies that specifically target neuroinflammatory pathways to combat these complex diseases. Hence, further research regarding the potential of TRF as a treatment for neuroinflammation is crucial. However, despite promising literature, studies exploring TRF’s effects on both cell and animal neuroinflammation models remain limited. This study focuses on evaluating the neuroprotective, anti-inflammatory and antioxidant effects of TRF by examining its effectiveness in reducing pro-inflammatory cytokines in microglia cells as well as its effects on cognitive impairment and gene expression levels of implicated downstream targets in animal-based neuroinflammation models.

## Methods

### Chemicals and reagents

The murine microglial cell line (BV2) was obtained from AcceGen, USA (#ABC-TC212S). Tocotrienol-Rich Fraction (TRF) was gifted by DavosLife, Malaysia with the bioactive components listed in Supplementary Table 1; whereas Lipopolysaccharide (LPS) from Escherichia coli O111: B4 (cat. no. tlrl-eblps) was purchased from InvivoGen, USA and streptozotocin (STZ) (cat. no. S0130) was purchased from Sigma-Aldrich USA. The high glucose Dulbecco’s modified Eagle medium (DMEM) (cat. no. 12–100-061) was acquired from Gibco by Life Technologies, USA and Fetal Bovine Serum (FBS) (cat. no. FBSEU500) was obtained from Tico, Europe. Accutase (cat. no. A6964) and Penicillin/Streptomycin (cat. no. PSG-B) was obtained from Sigma Aldrich, USA and Capricorn Scientific, Germany respectively. The 3-(4,5-dimethyl-thiazol-2-yl)-2,5-diphenyltetrazolium bromide (MTT) powder (cat. no. M2003) and 2′-7′-dichlorofluorescein diacetate (DCFDA) powder (cat. no. 410217) were obtained from Sigma-Aldrich, USA; whereas the Griess Reagent System (cat. no. G2930) was purchased from Promega, USA. Furthermore, the 1,1-diphenyl-2-picryl hydrazyl (DPPH) (cat. no. 281689), methanol (cat. no. V800258) and ascorbic acid (cat. no. PHR1008) were acquired from Sigma-Aldrich, USA. The QIAzol Lysis reagent (cat. no. 79306) was obtained from Qiagen, USA; whereas the High-Capacity cDNA Reverse Transcription Kit (cat. no. 4368814) was obtained from Applied Biosystem, USA and the Luna® Universal qPCR Master Mix (cat. no. M3003X) was purchased from New England Biolabs, USA. The qPCR primers TNF-α, IL-6, NFE2L2, RELA, BDNF genes were purchased from Integrated DNA Technologies (IDT), USA.

### BV2 cell culture

The BV2 murine microglial cells were cultured in complete Dulbecco’s Modified Eagle Medium (DMEM) supplemented with 10% Foetal Bovine Serum (FBS) and 1% Penicillin. Streptomycin. The cells were incubated in a humidified incubator at 37 °C and 5% CO2.

### Tocotrienol Rich-Fraction (TRF) stock solution preparation

For MTT and Greiss assays, a stock solution of TRF in absolute ethanol (100 mg/mL) was sterile filtered and stored at -80 °C prior to use. For DCFDA assay, a TRF stock with similar concentration was prepared with Dimethyl Sulfoxide (DMSO), sterile filtered and stored at -80 °C prior to use. For *in-vivo* treatment, the TRF stock solution was prepared every three days according to the treatment group and rat weight, dissolved in 1 mL of soy oil and kept in 4 °C prior to use.

### Cell viability assay

BV2 cells were seeded at 3.5 × 103 cells/well in a 96-well plate and incubated overnight, followed by treatment with various concentrations of TRF (100, 75, 50, 25, 10, 5 µg/mL) and incubated for 24 h. Subsequently the treatment solutions were removed and MTT reagent was added into the wells. The plate was incubated at 37 °C for 3 h before the purple formazan crystals were dissolve in DMSO. The absorbance was measured at 570 nm using the Tecan Infinite 200 Pro microplate reader.

### DPPH radical-scavenging assay

Various concentrations of TRF extract (1000, 500, 250, 100, 50, 25, 10, 5 µg/mL) were prepared in absolute ethanol and 50 µL of each concentration were added into a 96-well plate, followed by 250 µL of DPPH (5.9 mg/100 mL absolute ethanol). The reaction mixture was incubated at room temperature for 30 min and the absorbance values were measured at 517 nm using the Tecan Infinite M200 microplate reader and each experiment was conducted in triplicates. The scavenging activity (SA) percentage was calculated using the formula below and the IC50 was obtained. A calibration curve with ascorbic acid (1000, 500, 250, 100, 50, 25, 10, 5 µM) was plotted as comparison.

$$\begin{array}{*{20}c} {Scavenging } \\ {\quad Activity \left( \% \right) = } \\ \end{array} \frac{\begin{gathered} Absorbance\,of\,Negative \hfill \\ \quad Control - Absorbance\,of \hfill \\ \quad Treatment \hfill \\ \end{gathered} }{\begin{gathered} Absorbance\,of\,Negative \hfill \\ \quad Control \hfill \\ \end{gathered} } \times 100\%$$

### Nitric oxide quantification

BV2 cells were seeded at 3.5 × 103 cells/well in a 96-well plate and incubated overnight, followed by treatment with various concentrations of LPS (10, 5, 1, 0.5 µg/mL) and incubated for 48 h. Subsequently, 50 µL of the treatment solutions were transferred into a 96-well plate and the plate was incubated at room temperature for 10 min with 50 µL of sulphanilamide and N-1-napthylethylenediamine dihydrochloride (NED) solution respectively. The absorbance was measured at 525 nm using the Tecan Infinite 200 Pro microplate reader. This procedure was repeated as the seeded BV2 cells were treated with 5 and 10 µg/mL of TRF and incubated for 24 h. Subsequently the treatment solution was removed and 10 µg/mL of LPS was added. The plate was incubated for 48 h and the absorbance was similarly measured after incubations with sulphanilamide and NED solution for 10 min. A nitrite standard reference curve with nitric solution (100, 50, 23, 12.5, 6.25, 3.13, 1.56 µM) was plotted to calculate nitrite concentration.

### Reactive oxygen species analysis

BV2 cells were seeded at 3.5 × 103 cells/well in a black 96-well plate and incubated overnight, followed by treatment with 5 and 10 µg/mL of TRF and incubated for 24 h. Subsequently, the treatment solutions were removed and 0.1 µg/mL of LPS was added 15, followed by incubation for 24 h. The cells were washed once with phosphate- buffered saline (PBS) and 10 µM of DCFDA reagent was added. The plate was incubated at 37 °C for 45 min, the DCFDA reagent was subsequently removed and the cells were washed with PBS twice. The fluorescence intensity was measured with the Tecan Infinite 200 Pro microplate reader at excitation and emission wavelengths of 485 nm and 535 nm and the percentage of ROS production was calculated with the formula in below.

$$\% of\,ROS = \frac{\begin{gathered} Fluorescence\,of\,Treatment \hfill \\ \quad Groups - Blank \hfill \\ \end{gathered} }{\begin{gathered} Fluoresence\,of\,Vehicle \hfill \\ \quad Control - Blank \hfill \\ \end{gathered} }$$

### Rat maintenance

Locally bred adult male Sprague Dawley rats weighing between 250 and 400 g were obtained from the animal facility of Jeffery Cheah School of Medicine and Health Sciences, Monash University Malaysia. The rats were kept in separate cages under standard husbandry conditions with constant access to food and water, controlled room temperature of (22 ± 2 °C), in a stress-free and sanitary environment. The experiment protocols were approved and performed according to the approval of the Animal Ethics Committee Monash University Animal Research Platform (Project ID. 30073).

### In-vivo experimental group

Twenty rats were used in the study, prior to the experiment, the animals were acclimated to the surroundings for one week. The animals were then randomly assigned into 5 groups with 4 rats per group using simple randomization, according to the following:

- Group 1: Sham-control (Saline)
- Group 2: Negative Control (Saline + 3 mg/kg STZ)
- Group 3: TRF Low Dose (50 mg/kg TRF) + 3 mg/kg STZ
- Group 4: TRF Medium Dose (100 mg/kg TRF) + 3 mg/kg STZ
- Group 5: TRF High Dose (200 mg/kg TRF) + 3 mg/kg STZ

**Fig. 1:**
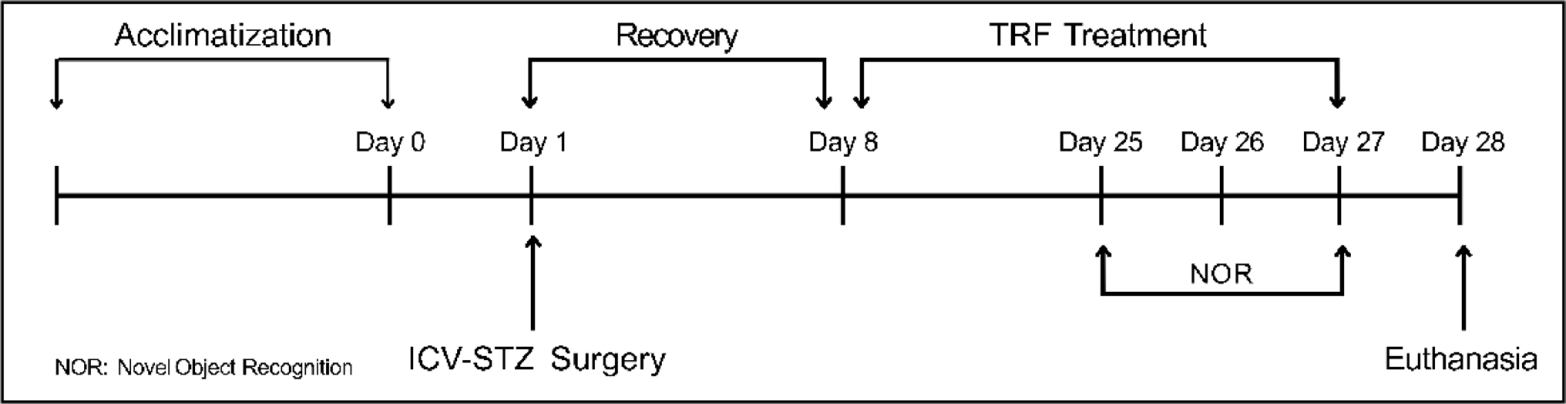
Schematic diagram of the *in-vivo* experimental design

The animals were subsequently subjected to intracerebroventricular (ICV) injection where saline or 3 mg/kg of STZ dissolved in saline were injected bilaterally into the brain. The rats were monitored for a week then subjected to a 28-day treatment period with TRF through oral dosing. Subsequently, a novel object recognition (NOR) study was conducted on days 25 to 27. After the 28th day of treatment, the rats were euthanized with an overdose of isoflurane, followed by decapitation and their hippocampus was extracted for subsequent gene expression analysis. Figure 1 depicts the experimental flow conducted.

### Intracerebroventricular (ICV) injection with streptozotocin (STZ)

An ICV surgery was performed according to Retinasamy et al. (2020), where the rats were initially anesthetized with 5% isoflurane and was subsequently kept at 2.5 to 3% throughout the surgery. During the surgery, the rat’s head was positioned and fixed on a stereotaxic frame. A midline sagittal incision was done on the scalp, followed by a burr hole drilled on both sides of the lateral ventricles according to the coordinates (0.08 mm posterior to bregma, 0.14 mm lateral to the sagittal suture, and 0.4 mm beneath the surface of the brain). Subsequently, an injection cannula was lowered into the burr holes formed and fresh STZ (3 mg/kg, 2 µL/injection site) or saline (2 µL per injection site) was delivered through the burr holes in the skull using a micro-injector unit. The injection cannula was left in place for 5 min then slowly removed from the scalp, the surgical cut was sutured close, and iodine solution was applied on the suture wound. After the surgery, the rats would be excluded if they don’t wake up after the surgery, or if they lose their appetite (no water or food intake) after waking up. Based on these criteria, no animals were excluded in this study.

### Novel object recognition

This study used the protocol from Mathiasen and DiCamillo (2010) with slight alterations. The equipment required includes an open field box made of black acrylic material (40 × 40 × 40 cm), a video camera (Sony Handycam HDR-CX405), a novel object and two familiar objects. The familiar objects were two similar specimen containers wrapped in black duct tape and a yellow screw cap whilst the novel object was a similar specimen container but with toy legos attached to the yellow screw cap. This experiment was conducted over 3 days under proper illuminated conditions with low-wattage light bulbs. During the first day, each rat was allowed to explore the open field box without any objects for 10 min for acclimatization, no motor activity was recorded during this phase. However, rats that had displayed severe freezing behavior were excluded from the experiment. The next day, each rat was placed in the open field with both familiar objects for 10 min and allowed to explore freely to familiarize with the objects. After 24 h, the rats undergo the test phase where one familiar object was swapped with a novel object, and the rat was left to explore for 10 min, this was repeated with the other familiar object to counterbalance. Throughout the familiarization and test phase, the rats’ exploration was recorded on video and the duration each rat spent to explore the familiar and novel object were later evaluated, rats that explored for less than 10 s were excluded from the experiment. During these two phases, exploration is defined with the rat pointing its nose at the object at a distance ≤ 2 cm, obvious vibrissae movement, sniffing, licking or rearing onto the object; whereas actions such as sitting on the object or close contact in which the nose is not directed at the object are not considered as exploration. To ensure proper exploration, the open field box and test objects were thoroughly cleaned with 70% ethanol after each test rat to prevent the rats from recognizing the objects based on scent trails. Furthermore, the experiment is carried out in a quiet environment to prevent disturbances and affect rats’ exploration The recognition index was calculated using the formula below, where TA and TB are time spent exploring familiar object A and novel object B respectively.

$$Recognition Index = \frac{TB}{{TA + TB}} \times 100$$

### Real time-quantitative polymerase chain reaction (RT-qPCR)

The total RNA from the rat’s hippocampus was extracted using phenol–chloroform extraction according to the protocol in Retinasamy et al. (2020). The hippocampal tissue was briefly homogenized in 1 mL of Qiazol solution and centrifuged at 13,000 rpm, 4 °C for 10 min. Subsequently, the supernatant was extracted and 200 µL chloroform was added, the mixture was centrifuged at 13,000 rpm, 4 °C for 15 min. The upper aqueous phase was then transferred to a new tube and 500 µL of isopropyl alcohol was added then centrifuged at 13,000 rpm, 4 °C for 10 min. The isopropyl alcohol was then removed, and the pellet was washed twice with 75% ethanol. The pellet was left to dry and 15 µL of RNase free water was added, the final RNA concentration was quantified using Nanodrop. Reverse transcription of 2000 ng total RNA into cDNA was performed using the High-Capacity cDNA Reverse Transcription Kit (Applied Biosystem, USA) according to the manufacturer protocol. Quantitative real-time PCR (qRT–PCR) was then carried out according to the Luna® Universal qPCR Master Mix kit protocol (New England Biolabs, USA) with the QuantStudio 5 Real-Time PCR System (Applied Biosystems, USA) to measure the mRNA expression levels of genes encoding TNF-α, IL-6, NFE2L2, RELA, BDNF and Glyceraldehyde-3-Phosphate Dehydrogenase (GAPDH) (Integrated DNA Technologies, USA) in the hippocampal samples. The specific sequences for each primer are listed in \* MERGEFORMAT Table 1. The relative mRNA expression level was calculated using the following formula and was normalized with the GAPDH gene.

$$\begin{array}{*{20}c} {Relative mRNA} \\ {\quad Expression\,Level} \\ \end{array} = 2^{{\left( \begin{subarray}{l} CT\,of\,References \\ \quad \quad - CT\,of\,Samples \end{subarray} \right)}}$$

**Table 1:** qPCR Primer Sequences

Target Gene	Forward Primer Sequence (5′-3′)	Reverse Primer Sequence (5′-3′)
TNF-α	CTTTCTAAGCGGAAATGAGTGC	GGAAGATAACCAGAGGCAACA
IL-6	CAGAGCAATACTGAAACCCTAGT	CCTTCTGTGACTCTAACTTCTCC
NFE2L2	CAGTGGATCTGTCAGCTACTC	CAAGCGACTCATGGTCATCTAC
RELA	GAGTTCCAGTACTTGCCAGAC	GACTCTTCTTCATGATGCTCTTG
BDNF	GACACATTACCTTCCAGCATCT	GCAACCGAAGTATGAAATAACCA
GADPH	AACCCATCACCATCTTCCAG	CCAGTAGACTCCACGACATAC

### Statistical analysis

The statistical analysis was conducted using GraphPad Prism 8.0 software. The data obtained from *in-vitro* analysis were depicted in the form of mean ± standard deviation (mean ± SD) and the data obtained from the *in-vivo* analysis were expressed in the form of mean ± standard error (mean ± S.E.M). The statistical differences between groups were obtained using one-way ANOVA, followed by Dunnett’s multiple comparison post hoc test with a *p* value of < 0.05 considered as statistically significant. No test was conducted for normality and outliers.

## Results

### Cytotoxic effects of TRF on BV2 cells

MTT assay was performed to obtain the non-cytotoxic concentration of TRF to be used as treatment in subsequent assays. As seen in .

**Fig. 2:**
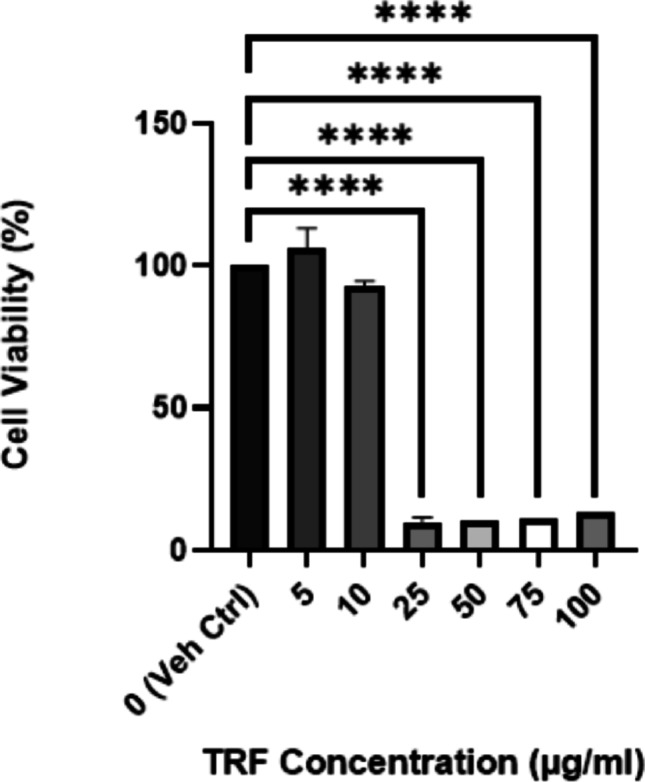
Cell viability percentage of BV2 cells was determined by MTT assay after 24 h exposure to 0, 5, 10, 25, 50, 75, 100 µg/mL. All values are presented in the form of mean ± SD from two biological replicates with three technical replicates in each group. Data points obtained for each group are presented and each concentration group is compared with the vehicle control (veh. ctrl) which consists of 1% absolute ethanol. The statistical differences are presented as ****p < 0.0001 versus vehicle control group

Figure 2, after TRF treatment with concentrations ranging from 0 (vehicle control) to 100 µg/mL for 24 h, it was found that when compared with the vehicle control, the concentrations 5 and 10 µg/mL had no significant difference on BV2 cell viability (*p* > 0.05). Hence, these two concentrations (5 and 10 µg/mL) were used for treatment in subsequent Griess and DCFDA assays to evaluate the dose–response effects of TRF on LPS-induced oxidative stress.

### Antioxidant properties of TRF

DPPH radical scavenging assay was conducted to evaluate the antioxidant properties of TRF. It was found that TRF has a slightly weaker antioxidant capacity (IC_50_ value of 6.61 µg/mL) compared to ascorbic acid (IC_50_ of 2.85 µg/mL) (Table 2). The reported IC_50_ value is the concentration of TRF, and ascorbic acid required to reduce the absorbance of the DPPH radical at 517 nm to half the initial value after 30 min at room temperature.

**Table 2:** Antioxidant properties of TRF

Compound	IC_50_ ± SD (µg/mL)
Ascorbic Acid	2.85 ± 0.05
Tocotrienol Rich Fraction (TRF)	6.61 ± 0.25

### Effects of TRF on LPS-induced NO and ROS production in BV2 cells

**Fig. 3:**
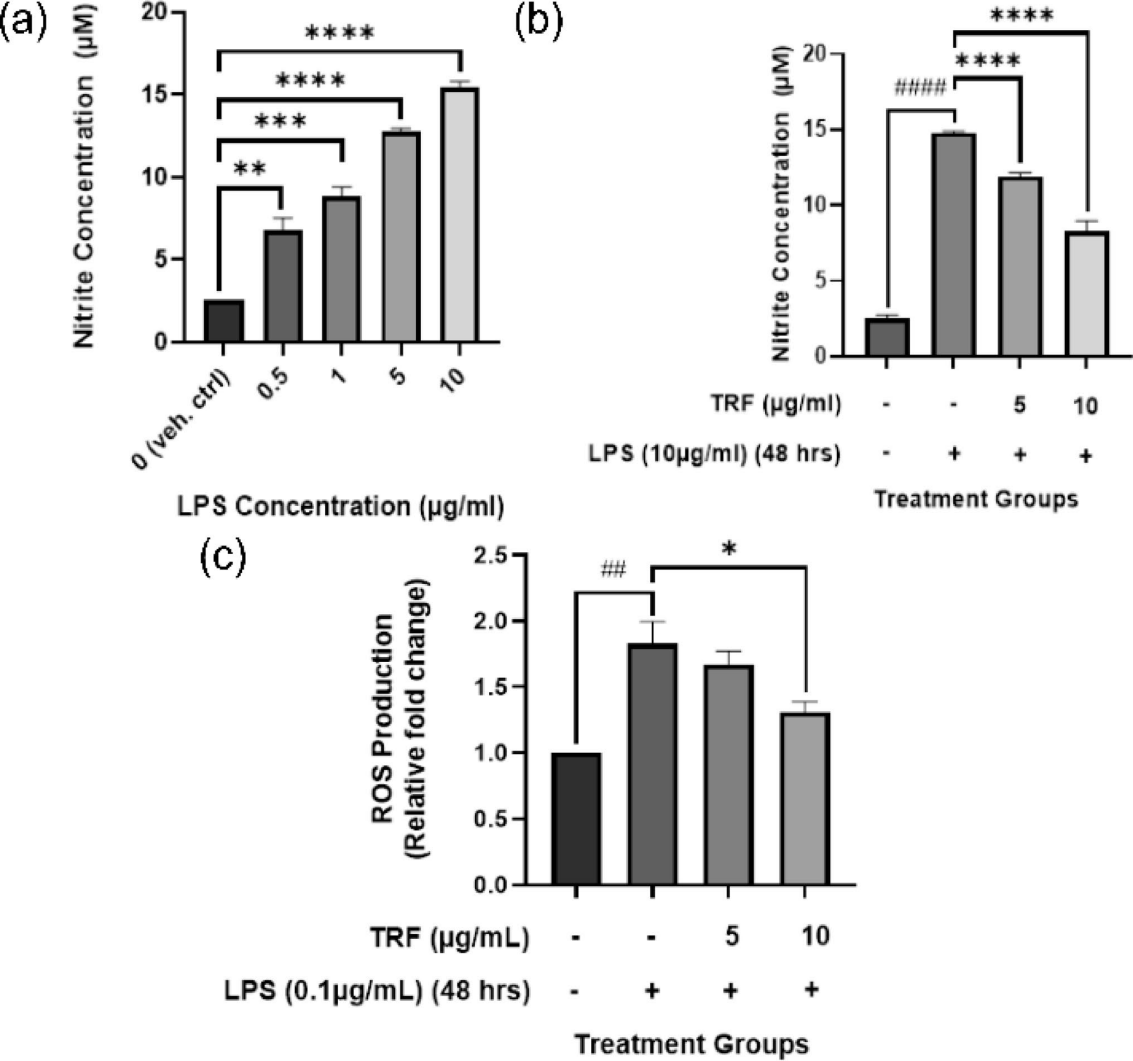
Effects of TRF on LPS-induced NO and ROS production in BV2 cells. **a** NO production (µM) in BV2 cells after exposure to 0, 0.5, 1, 5, 10 µg/mL LPS for 48 h. The statistical differences are presented as **p < 0.01, ***p < 0.001 and ****p < 0.0001 compared with the vehicle control (veh. ctrl). **b** NO production (µM) in BV2 cells after pretreatment with 5 and 10 µg/ml TRF for 24 h, followed by exposure to 10 µg/mL LPS for 48 h. The statistical differences are presented as ####*p* < 0.0001 compared with vehicle control consisting of 1% absolute ethanol only, without TRF and LPS treatment; ****p < 0.0001 compared with the positive control consisting of 10 µg/mL LPS only. **c** ROS production in BV2 cells after pretreatment with 5 and 10 µg/mL TRF for 24 h, followed by exposure to 0.1 µg/mL LPS for 24 h. The statistical differences are presented as ##*p* < 0.01 compared with the vehicle control consisting of 0.2% DMSO only, without TRF and LPS treatment; *p < 0.05 compared with the positive control consisting of 0.1 µg/mL LPS only. All values are presented in the form of mean ± SD from two biological replicates with three technical replicates in each group

Griess assay was used to determine the effects of TRF on NO production induced by LPS. After exposure to LPS (0.5, 1, 5, 10 µg/mL) for 48 h (Retinasamy et al. 2024), it was found that 10 µg/mL of LPS produced the highest concentration of NO (Fig. 3a). The NO production is directly proportional to LPS concentration used, indicating a dose-dependent pattern. Subsequent assay uses 10 µg/mL of LPS as an inducer to evaluate the anti-inflammatory effects of TRF. Based on the results in Fig. 3(b), both TRF concentrations (5 and 10 µg/mL) significantly decreased NO production in BV2 cells compared to the positive control and the anti-inflammatory effects of TRF was found to be dose dependent. The results showed that 10 µg/mL of TRF reduced NO production by 6.55 µM (*p* < 0.0001) while 5 µg/mL of TRF decreased NO production in BV2 cells by 2.90 µM (*p* < 0.0001).

DCFDA assay was carried out to evaluate the antioxidant effects of TRF (5 and 10 µg/mL) on ROS production in BV2 cells. From Fig. 3(c), it can be observed that after exposure to 0.1 µg/mL LPS for 24 h (Velagapudi et al. 2014), the BV2 cells pretreated with 10 µg/mL TRF showed a significant decrease in ROS production with a reduction of 51.84% compared to the LPS-treated group (*p* < 0.05). However, ROS production with BV2 cells pretreated with 5 µg/mL of TRF did not show any significant reduction (*p* > 0.05).

### Effects of TRF on cognitive impairment in STZ-induced rats

**Fig. 4:**
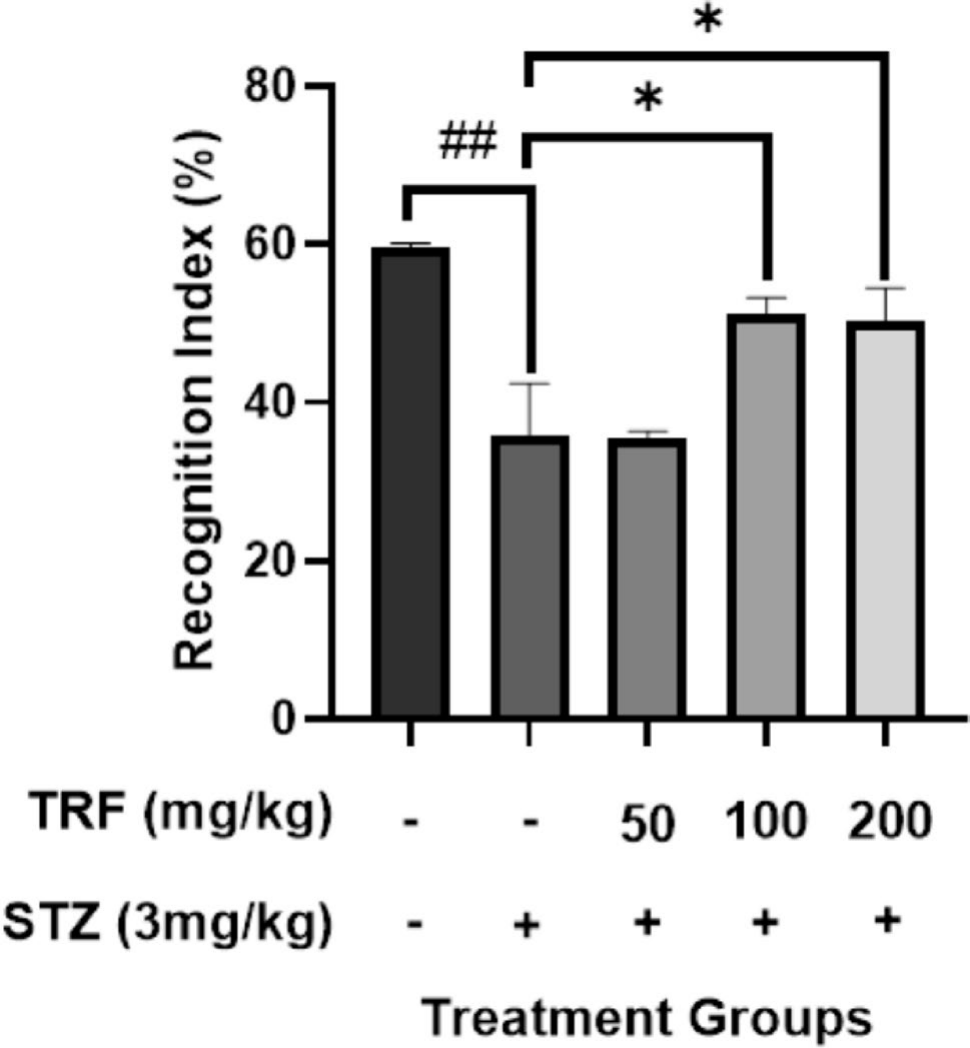
Effects of TRF on cognitive impairment in STZ induced rats. Novel object recognition (NOR) was carried out for behaviour analysis and the recognition index was calculated according to object exploration duration. The recognition index in the negative control (3 mg/kg STZ) was compared with the saline control, whereas the changes in recognition index in the treatment groups (50, 100, 200 mg/kg TRF) was compared with the negative control (3 mg/kg STZ). The data above is presented in the form of mean ± SEM, n = 4. The statistical differences are presented as ##*p* < 0.01 compared with the saline control and *p < 0.05 compared with the negative control

Novel Object Recognition (NOR) was conducted to evaluate the effects of TRF on cognitive impairment in STZ-induced rats, where the recognition index was calculated based on object exploration duration. Based on the results in Fig. 4, the negative control group (3 mg/kg STZ) had a significant decrease in recognition index of 23.80% when compared with the saline control (*p* < 0.01). Although the low dose group (50 mg/kg TRF) did not have any significant changes in recognition index compared to the negative control (3 mg/kg STZ), the middle dose (100 mg/kg TRF) and high dose groups (200 mg/kg TRF) had a significant increase in recognition index of 15.45% and 14.22% respectively (*p* < 0.05) when compared with the negative control (3 mg/kg STZ).

### Effects of TRF on pro-inflammatory markers in STZ-induced rats

**Fig. 5:**
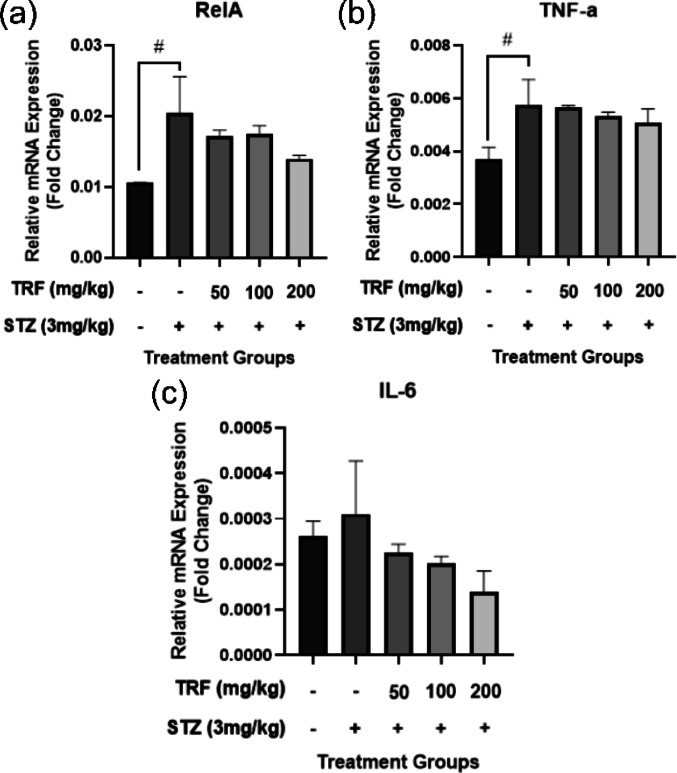
Effects of TRF on neuroinflammatory markers in STZ-induced rats. The relative mRNA expression of **a** RelA, **b** TNF-α and **c** IL-6 were quantified using RT-qPCR. The changes in mRNA expressions levels in the negative control (3 mg/kg STZ) were compared with the saline control, whereas the changes in mRNA expression level in the treatment groups (50, 100, 200 mg/kg TRF) were compared with the negative control (3 mg/kg STZ). The data above is presented in the form of mean ± SEM, n = 4. The statistical differences are presented as #p < 0.05 compared with the saline control and *p < 0.05 compared with the negative control (3 mg/kg STZ)

Given that TRF reduced LPS-induced NO production in BV2 cells, RT-qPCR was conducted to further evaluate the effects of TRF on the mRNA expression levels of pro-inflammatory markers RelA, TNF-α and IL-6 in STZ-induced rats. Based on Fig. 5a, b and c, a general upregulation in RelA, TNF-α and IL-6 expression was observed between the negative control (3 mg/kg STZ) group compared with the saline control. Overall, there was a downregulation in mRNA expression for RelA, TNF-α and IL-6 in all TRF treatment groups. However, the trend in downregulation in TNF-α was least pronounced compared to RelA and IL-6 whereas IL-6 had the largest downregulating trend with its treatment groups having a lower mRNA expression compared to the saline and negative control (3 mg/kg STZ). Unfortunately, despite the general downregulating trend found, the gene expression level of RelA, TNF-α and IL-6 did not exhibit any significant downregulation compared to the negative control (3 mg/kg STZ) (*p* > 0.05).

### Effects of TRF on oxidative stress markers in STZ-induced rats*.*

**Fig. 6:**
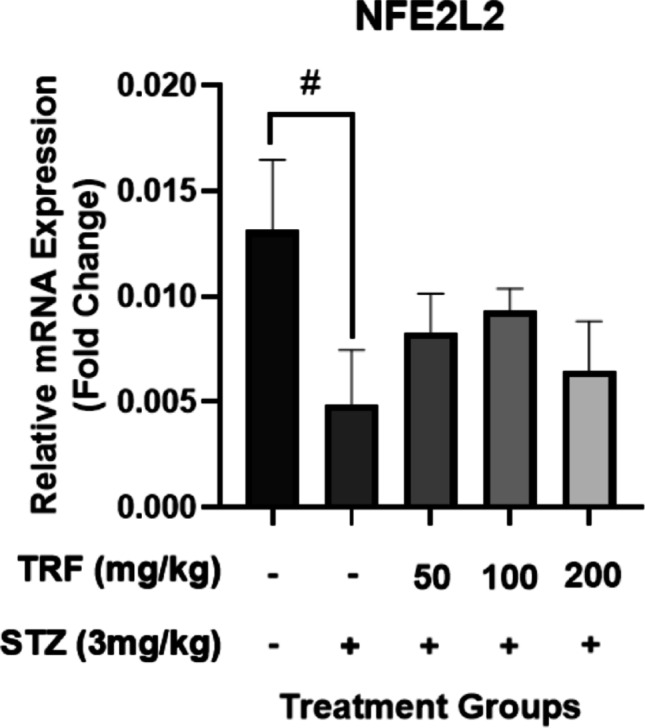
Effects of TRF on oxidative stress marker in STZ-induced rats. The relative mRNA expression of NFE2L2 were quantified using RT-qPCR. The changes in mRNA expressions levels in the negative control (3 mg/kg STZ) were compared with the saline control, whereas the changes in mRNA expression level in the treatment groups (50, 100, 200 mg/kg TRF) was compared with the negative control (3 mg/kg STZ). The data above is presented in the form of mean ± SEM, n = 4. The statistical differences are presented as #*p* < 0.05 compared with the saline control

Similarly, as TRF reduced LPS-induced ROS production in BV2 cells, RT-qPCR was conducted to further evaluate the effects of TRF on the oxidative stress marker NFE2L2 in STZ-induced rats. Based on Fig. 6, there was a downregulation in mRNA expression level between the negative control (3 mg/kg STZ) and the saline control (*p* < 0.05) whereas there is an overall upregulation in mRNA expression in TRF treatment groups. It can be observed in Fig. 6 that the middle dose (100 mg/kg TRF) group had the greatest increase in mRNA expression levels compared with the negative control (3 mg/kg STZ), followed by a decrease as the TRF concentration increases to high dose (200 mg/kg). However, despite the upregulating trend in mRNA expression levels between the treatment groups (50, 100, 200 mg/kg TRF) and the negative control (3 mg/kg STZ) the results were not significant (*p* > 0.05).

### Effects of TRF on synaptic plasticity and neurogenesis in STZ-induced rats

**Fig. 7:**
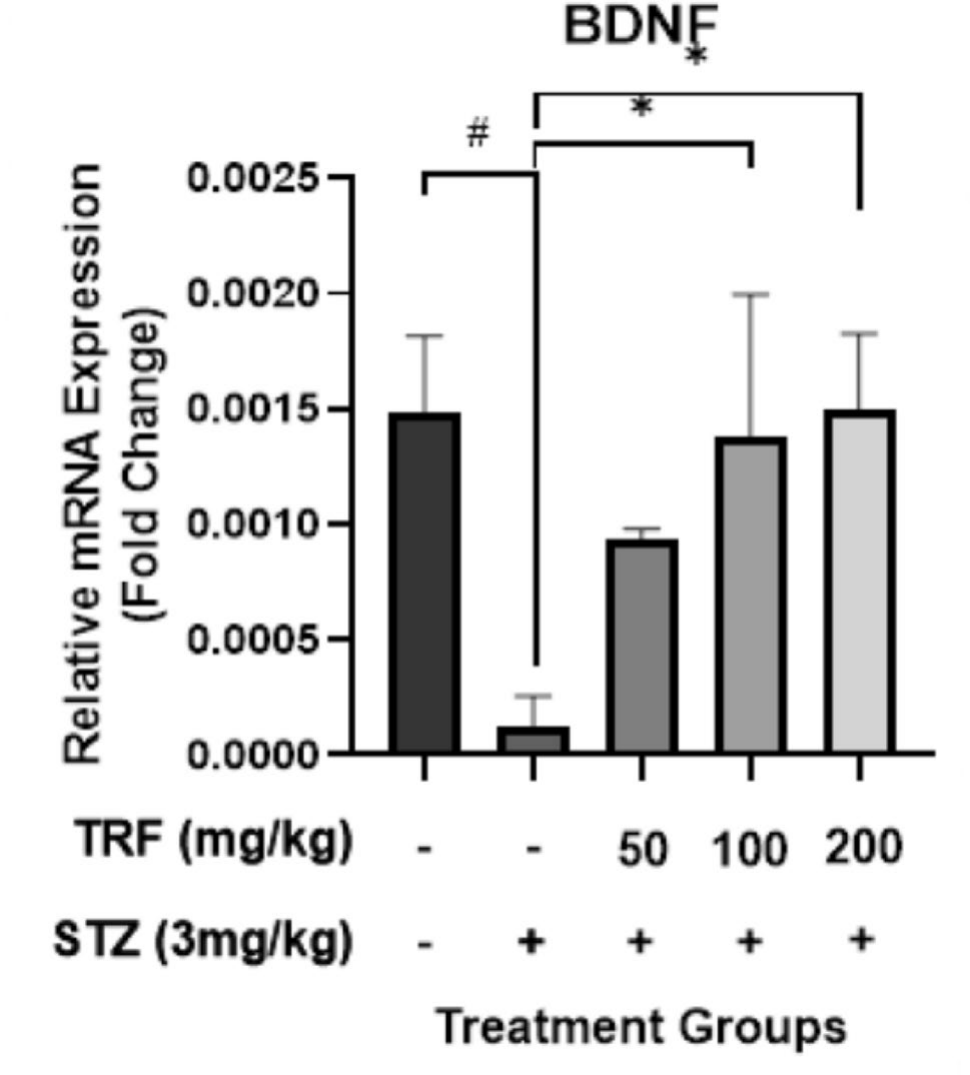
Effects of TRF on synaptic plasticity and neurogenesis in STZ induced rats. The relative mRNA expression of BDNF were quantified using RT-qPCR. The changes in mRNA expressions levels in the negative control (3 mg/kg STZ) were compared with the saline control, whereas the changes in mRNA expression level in the treatment groups (50, 100, 200 mg/kg TRF) was compared with the negative control (3 mg/kg STZ). The data above is presented in the form of mean ± SEM, n = 4. The statistical differences are presented as #*p* < 0.05 compared with the saline control and *p < 0.05 compared with the negative control (3 mg/kg STZ)

RT-qPCR was conducted to evaluate the effects of TRF on synaptic plasticity and neurogenesis in STZ-induced rats as it quantifies the mRNA expression level of BDNF. Based on Fig. 7, there is a significant downregulation between the saline control and the negative control (3 mg/kg STZ) (*p* < 0.05). There was a general upregulation in mRNA expression level following TRF treatment in STZ-induced rats. However, only the middle dose (100 mg/kg TRF) and high dose (200 mg/kg TRF) treatment groups showed significant results when compared with the negative control (3 mg/kg STZ) (*p* < 0.05). The upwards trend in mRNA expression demonstrated a dose-dependent pattern on BDNF when treated with various concentrations of TRF.

## Discussion

Neuroinflammation is a vicious cycle where a persistent inflammatory stimulus causes synaptic dysfunction and neuronal damage. It usually exacerbates the development and progression of neurodegenerative diseases such as AD, PD and HD (Adamu et al. 2024; Kwon and Koh 2020). The persistent inflammatory stimulus overstimulates the glial cells (microglial and astrocytes), activating the NF-κB pathway and resulting in the excessive production pro-inflammatory cytokines, nitric oxide (NO) and reactive oxygen species (ROS). The accumulation of NO and ROS causes nitrosative and oxidative stress, which contribute to neuroinflammation and cognitive decline, specifically leaning and spatial memory impairment due to the neuroinflammatory response and oxidative stress (Rosi et al. 2005; Retinasamy et al. 2020; Abdallah et al. 2021; Agrawal et al. 2010).

In this study, BV2 cells were used to investigate the effect of TRF on neuroinflammation (Li et al. 2023). It was found that high concentrations of TRF (> 10 µg/mL) possessed cytotoxic effects which are consistent with the observation reported by Ng and Ko (2012). Thus, 10 and 5 µg/mL (non-toxic concentrations) of TRF were selected to evaluate its dose-dependent effects on NO and ROS reduction. The cytotoxicity is likely related to the presence of *γ*-tocotrienol in the compound, which although protective at a low concentration (< 10 µM), can exhibit its cytotoxic effects by promoting apoptosis through caspace-3 activation at higher concentrations (Abd Manan et al. 2012). This hypothesis is supported by previous studies where cytotoxic effects have been observed in primary astrocytes cultures with 200 µM of *γ*-tocotrienol (Mazlan et al. 2006) and primary neuron cultures with 100 µM of *γ*-tocotrienol (Sue Mian et al. 2009). Furthermore, TRF dosages (50, 100 and 200 mg/kg) utilized in the *in-vivo* analysis was selected based on the preliminary disease-deciding study conducted by Retinasamy et al. (2020) and are within the safety limits for human consumption.

Lipopolysaccharide (LPS) induces neuroinflammation in BV2 cells by activating the NF-κB pathway, which increases NO and ROS levels while preventing ROS clearance by supressing antioxidative enzyme, resulting in oxidative stress (Kim et al. 2014; Subedi et al. 2021; Li et al. 2010). Similarly, intracerebroventricular (ICV) surgery of streptozotocin (STZ) into the rodent’s brain induces neuroinflammation, oxidative stress and biochemical alterations making it a suitable inducer for studying the early pathophysiological changes in neurodegenerative diseases (Kamat 2015; Retinasamy et al. 2020). It activates glial cells and triggers NF-κB pathway, causing excessive production of pro-inflammatory cytokines, NO and ROS, which forms a positive feedback loop that mimics neuroinflammation environment (Adamu et al. 2024; Rai et al. 2014; Omer et al. 2023). This can be reflected by the decreased in recognition index observed in this study, aligning with Gáspár et al. (2022), indicating that STZ induces cognitive impairment.

The activation of the NF-κB pathway is an essential therapeutic target for neuroinflammation, where the effects can be observed with changes in production of inflammatory products NO, ROS and pro-inflammatory cytokines. NF-κB is a transcription factor composed of 5 subunits: NF-κB1 (p50), NF-κB2 (p52), RelA (p65), RelB and c-Rel. Among these, RelA predominantly regulates the transcription of pro-inflammatory genes in microglia (Kaminska et al. 2016). Hence, the expression levels of RelA were measured to evaluate the anti-inflammatory effects of TRF on NF-κB pathway activation. In the current pilot study, TRF treatment dose-dependently downregulated RelA expression levels *in-vivo* when compared to the significant upregulation in the ICV-STZ group. This shows that TRF potentially inhibits NF-κB pathway activity, which aligns with previously reported study Ng and Ko (2012).

According to Ang et al. (2025), TRF potentially inhibits the NF-κB signalling pathway by blocking the degradation of IκB. IκB is an inhibitory protein that is bound to NF-κB, and which sequesters its activity in the cytoplasm (Perkins 2007). When neuroinflammation occurs, IκB is cleaved from NF-κB which allows the free activated NF-κB to translocate to the nucleus and in turn activates the NF-κB signalling pathway (Singh and Singh 2020). As TRF was shown to prevent IκB degradation, it likely retains NF-κB in the cytoplasm and inhibits the activation of the NF-κB signalling pathway and the transcription of pro-inflammatory mediators (Wang et al. 2015; Ahn et al. 2007). The downregulated RelA expression levels correlates with the decrease in TNF-α and IL-6, corroborating TRF’s potential to inhibit the NF-κB pathway, which reduces the production of inflammatory cytokines. This observation also aligns with those reported by Lü et al. (2022), where TRF successfully reduced TNF-α and IL-6 expression levels in pulmonary fibrosis-induced rats.

Notably, TNF-α showed a milder downregulation than IL-6, similarly observed in Wang et al. (2017). This may be attributed to the NF-κB pathway being the primary regulator of TNF-α. As TRF inhibits the NF-κB pathway early in the neuroinflammatory cycle, this early disruption could establish a lower baseline level of TNF-α, consequently limiting the extent of further downregulation upon treatment (Wu and Zhou 2010). In contrast, the greater downregulation observed in IL-6 suggests that its expression could be affected not only by the NF-κB pathway, but also through other signalling mechanisms such as the Janus kinase/signal transducer and activator of transcriptions (JAK/STAT) pathway (Ciryam et al. 2023; Kern et al. 2018). Hence, the inhibition activity of TRF on the NF-κB pathway may have limited effects on IL-6 expression, potentially contributing to a higher baseline level of IL-6, and, consequently, a more pronounced downregulation trend. However, as this study focused solely on the effects of TRF on the NF-κB pathway, further investigations should assess biomarkers specific to the JAK/STAT pathway such as CXCL10 and CXCL9 (Hu et al. 2021).

As TRF potentially inhibits the NF-κB pathway and supresses the production of pro-inflammatory cytokines such as TNF-α and IL-6, it may disrupt the positive feedback loop responsible for excessive production of proinflammatory cytokines, NO and ROS. This in turn could help in alleviating neuroinflammation by reducing oxidative and nitrosative stress caused by the accumulation of NO and ROS, hence mitigating synaptic dysfunction and neuronal damage. The excessive production of NO plays a significant role in the exacerbation of neurodegenerative diseases as contributes to neuronal damage and synaptic dysfunction (Liy et al. 2021). It is primarily synthesized by activated microglial cells through inducible nitric oxide synthases (iNOS) by converting L-arginine to NO and L-citrulline in the presence of NADPH_2_ and O_2_ (Subedi et al. 2021; Yuste et al. 2015). Excessive NO production leads to the formation of reactive nitrogen species, resulting in nitrosative stress and subsequent apoptosis (Liy et al. 2021). TRF prevents NO accumulation by inhibiting the NF-κB pathway and also the iNOS activity, which is critical in the progression of neuroinflammation. Kummer et al. (2011) has previously demonstrated that iNOS knockout mice displayed superior memory function when compared with controls.

In addition, ROS overproduction during neuroinflammation will induce oxidative stress in the brain. This usually oxidizes brain proteins, nucleic acids and lipids, which will exacerbate the development of neurodegenerative diseases (Gao et al. 2014). The potent antioxidant activity of TRF can be attributed to the presence of δ-, γ—and α-tocotrienols, which enhance cellular uptake and allow it to act as a strong free radical scavenger (Muid et al. 2013; Palozza et al. 2006; Nor Azman et al. 2018). The potent free radical scavenging activity of TRF was demonstrated in this study, with a low IC_50_ value of 6.61 µg/mL This was further studied with LPS-induced BV2 cells, where 10 µg/mL significantly decreased the produced ROS levels.

Additionally, TRF upregulated NFE2L2 expression levels, which is a gene encoding the transcription factor Nuclear Factor Erythroid 2-related factor 2 (Nrf2). Nrf2 is a part of the Nrf2-Antioxidant Response Element (ARE) pathway where it is responsible for the regulation of antioxidant enzymes expression levels that allow the Nrf2-ARE pathway to mitigate neuroinflammation by removing ROS (Shal et al. 2018; Singh et al. 2021; Nguyen et al. 2009). NFE2L2 upregulation after TRF treatment suggests that TRF potentially activates this pathway, releasing Nrf2 from the inhibitory Nrf2 Kelch Protein (Keap1). This increases the expression of antioxidant enzymes such as catalase (CAT) and superoxide dismutase (SOD), increasing ROS clearance and preventing oxidative stress (Singh et al. 2021; Yoon et al. 2016). Notably, it was previously mentioned in Saha et al. (2020) that Nrf2 activation also inhibits NF-κB-mediated transcription for pro-inflammatory cytokines genes. This was observed in this study, with a downregulation in RelA, TNF-α and IL-6 expression levels in contrast with the upregulation in NFE2L2 expression levels.

Neuroinflammation-induced cognitive decline was evident in this study, where a significant decrease in recognition index was observed in ICV-STZ treated rodents in the NOR assay. This was consistent with results from Gáspár et al. (2022) indicating that STZ induces spatial and learning memory impairment. Based on previous literature, brain-derive neurotrophic factor (BDNF), a neurotrophin that is strongly related with synaptic plasticity, neuronal survival and neurogenesis, is a crucial factor in cognitive decline (Agrawal et al. 2010; Liy et al. 2021). In this study, TRF treatment increased BDNF expression levels correlating with an increase in recognition index, aligning with prior literature in BDNF expression in mouse hippocampal HT22 neuronal cells and AβPP/PS1 double transgenic mice (Rui et al. 2024; Durani et al. 2018). The correlation between BDNF upregulation and recognition index indicates the potential of TRF as a treatment for cognitive decline, specifically spatial and learning memory impairment. This can be supported by previous literature where a BDNF blockage was associated with impaired spatial memory and learning, whereas an increase in BDNF mRNA in the hippocampus is involved in improved spatial memory and learning (Radiske et al. 2017; Mizuno et al. 2000). As the expression levels of BDNF are heavily influenced by the overproduction of pro-inflammatory cytokines and ROS in neuroinflammation (Retinasamy et al. 2024). The findings in this study suggests that as TRF potentially inhibits the NF-κB pathway and activate the Nrf2-ARE pathway, its neuroprotective effects to decrease cognitive decline in neuroinflammation may involve preventing the overproduction of pro-inflammatory cytokine and ROS production.

In conclusion, TRF demonstrated its potential to mitigate neuroinflammation due to its neuroprotective, anti-inflammatory and anti-oxidative properties. It exhibited its free radical scavenging activity as it significantly reduced ROS levels whilst decreasing NO production in LPS-induced BV2 cells. This prevents oxidative and nitrosative stress, decreasing synaptic dysfunction and neuronal damage in neuroinflammation. Among others, TRF could act on the NF-κB and Nrf2-ARE pathways in ICV-STZ induced neuroinflammation Sprague Dawley rats. Furthermore, the reduction in pro-inflammatory cytokines and oxidative stress may potentially improve neuroinflammation-induced cognitive decline by upregulating BDNF expression levels, which increases synaptic plasticity, neuronal survival and neurogenesis, improving in spatial and learning memory impairment as seen with an increase in recognition index. Although promising, further investigations including studies with larger sample sizes and other relevant mechanistic validations are necessary to substantiate the potential of TRF in combating neuroinflammation.

## Supplementary Information

Below is the link to the electronic supplementary material.

Supplementary Material 1

## Data Availability

The data that support the findings of this study are available upon request.
