# Supplementary material for: Exploring the role of tocotrienol-rich fraction (TRF) in ameliorating neuroinflammation
Source: Inflammopharmacology. 2026 Apr 20;34(6):4117–30. doi: 10.1007/s10787-026-02249-8 (PMC13275581; doi:10.1007/s10787-026-02249-8)
Supplement: Supplementary file 1 — Supplementary Material 1 [file 10787_2026_2249_MOESM1_ESM.docx]

**Exploring the role of Tocotrienol-rich fraction (TRF) in ameliorating neuroinflammation**

**Jing Yi Tan^a^, Thaarvena Retinasamy^b*^, Vanessa Lin Lin Lee^b^, Ammu Kutty Radhakrishnan^c^ and Keng Yoon Yeong^a*^**

*^a^School of Science, Monash University Malaysia Campus, Jalan Lagoon Selatan, Bandar Sunway, 47500, Selangor, Malaysia.*

*^b^Neuropharmacology Research Laboratory, Jeffrey Cheah School of Medicine and Health Sciences, Monash University Malaysia, Bandar Sunway 47500, Selangor, Malaysia*

*^c^Food as Medicine Research Strength, Jeffrey Cheah School of Medicine and Health Sciences, Monash University Malaysia, Bandar Sunway, 47500 Petaling Jaya, Selangor, Malaysia*

Corresponding authors:

Keng Yoon Yeong - School of Science, Monash University Malaysia, Bandar Sunway 47500, Selangor, Malaysia

Tel.: +60 3 5514 6102; Email: [yeong.kengyoon@monash.edu](mailto:yeong.kengyoon@monash.edu)

Thaarvena Retinasamy - Neuropharmacology Research Laboratory, Jeffrey Cheah School of Medicine and Health Sciences, Monash University Malaysia, Bandar Sunway 47500, Selangor, Malaysia

Email: [thaarvena.retinasamy@monash.edu](mailto:thaarvena.retinasamy@monash.edu)


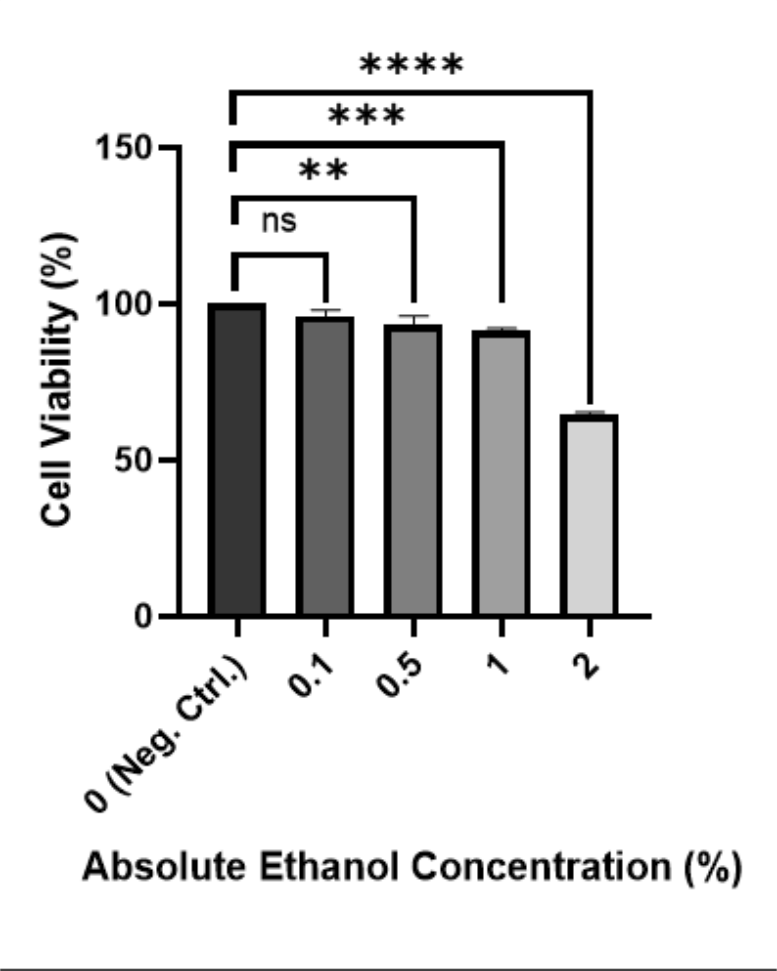


**Suppl. Fig. S 1:** Cell Viability percentage of BV2 cells was determined by MTT assay after 24 hours exposure to 0, 0.1, 0.5, 1 and 2% of absolute ethanol (AE). All values are presented in the form of mean percentage ± SD from three biological replicates with four technical replicates in each group; and each concentration group is compared with the negative control (Neg. Ctrl) which is media only. The statistical differences are presented as ***p*<0.05, ****p*<0.001 and *****p*<0.0001 compared with the negative control

**
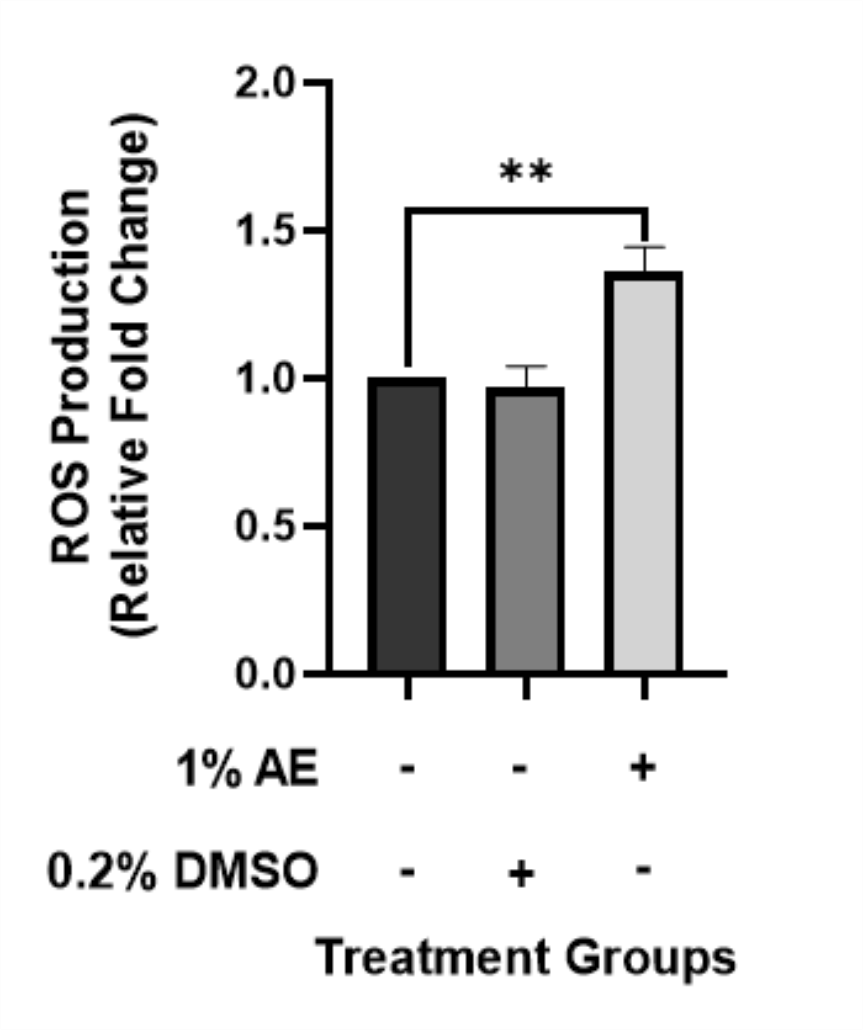
**

**Suppl. Fig. S 2:** ROS production from BV2 cells after exposure to either 1% absolute ethanol or 0.2% DMSO without inducer for 24 hours. The data showed the influence of vehicle control on ROS production in LPS-induced BV2 cells. All values are presented in the form of mean percentage ± SD from three biological replicates with three technical replicates in each group. The statistical differences are presented as ***p*<0.01 compared with the negative control which only consists of media.

**Supplementary Table 1:** **List of bioactive components in DavosLife Tocotrienol-Rich Fraction (TRF)**

| Component | Percentage (%) |
| --- | --- |
| α-tocopherol | 27.5 |
| α-tocotrienol | 26.1 |
| ß-tocotrienol | 2.6 |
| γ-tocotrienol | 30.6 |
| δ-tocotrienol | 9.7 |
| α-tocomonoenol | 3.3 |

*The bioactive components present in the DavosLife Tocotrienol-Rich Fraction (TRF) was provided by Davoslife, Malaysia using HPLC
